# Long‐Term Outcomes of Guided Internet‐Based Cognitive Behavioral Therapy for Anorexia Nervosa: 3‐Year Follow‐Up

**DOI:** 10.1002/brb3.70731

**Published:** 2025-09-08

**Authors:** Sayo Hamatani, Kazuki Matsumoto, Jumpei Takahashi, Eiji Shimizu, Yoshiyuki Hirano, Yoshifumi Mizuno

**Affiliations:** ^1^ Research Center for Child Mental Development University of Fukui Fukui Japan; ^2^ Division of Developmental Higher Brain Functions United Graduate School of Child Development University of Fukui Fukui Japan; ^3^ Department of Child and Adolescent Psychological Medicine University of Fukui Hospital Fukui Japan; ^4^ Research Center for Child Mental Development Chiba University Chiba Japan; ^5^ Division of Clinical Psychology, Kagoshima University Hospital, Research and Education Assembly Medical and Dental Sciences Area Kagoshima University Kagoshima Japan; ^6^ Department of Psychiatry Aoba Municipal Hospital Chiba Japan

**Keywords:** anorexia nervosa, cognitive behavioral therapy, Cost‐effectiveness, follow‐up study, guided internet‐based cognitive behavioral therapy, Japan

## Abstract

**Background:**

Guided internet‐based cognitive behavioral therapy (ICBT) provides an accessible alternative treatment for anorexia nervosa (AN), showing initial feasibility and effectiveness in the short term. However, limited research has explored its long‐term outcomes in outpatient settings.

**Objective:**

This study investigated the long‐term outcomes and cost‐effectiveness of guided ICBT in women with AN who were receiving outpatient treatment.

**Methods:**

This study involved a 3‐year follow‐up of 11 adult women with AN who received guided ICBT in a previous trial. The Eating Disorder Examination Questionnaire (EDE‐Q) and body mass index (BMI) were used as outcomes to assess the severity of AN. A repeated‐measures ANOVA was conducted to assess differences in mean outcomes at baseline, post‐intervention, and two follow‐up time points. The incremental cost‐effectiveness ratio was calculated to estimate the cost‐effectiveness of this intervention, using quality‐adjusted life years (QALYs), derived from the EuroQol 5 Dimension 5‐Level (EQ‐5D‐5L), and costs from publicly available data from the Japanese healthcare system.

**Results:**

From baseline, EDE‐Q global scores significantly decreased at the 1‐year (*d* = −0.72) and 3‐year follow‐ups (*d* = −0.65), while BMI significantly increased at the 1‐year (*d* = 0.66) and 3‐year follow‐ups (*d* = 0.98). The estimated incremental cost‐effectiveness ratio was notably low, at approximately JPY 111,000 (US$725) per year.

**Conclusion:**

These preliminary results suggest that guided ICBT may be effective in the long term for outpatients with AN. Since the threshold has been JPY 5 million in Japan, the current estimated incremental cost‐effectiveness ratio suggests that providing guided ICBT to outpatients with AN is likely to be cost‐effective.

## Graphical abstract

Guided internet‐based cognitive behavioral therapy (ICBT) for anorexia nervosa showed improvements in eating disorder symptoms and BMI at 1‐ and 3‐year follow‐ups. The intervention demonstrated strong cost‐effectiveness, with an incremental cost‐effectiveness ratio of JP¥111,000 (US$725) per QALYwell below Japan's threshold for healthcare interventions.

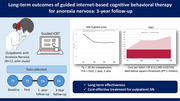

## 1 Introduction

Anorexia nervosa (AN) is a mental disorder characterized by a desire for thinness and an intense fear of gaining weight (American Psychiatric Association 2022). The lifetime prevalence of AN is up to 4% in women and 0.3% in men (van Eeden et al. 2021). Comorbid mental disorders such as major depressive disorder and anxiety disorders are highly prevalent in individuals with AN (Hambleton et al. 2022). The suicide rate among individuals with AN is 31 times higher than that in the general population (Preti et al. 2011). Therefore, those specialized treatments are essential for patients with AN.

Face‐to‐face cognitive behavioral therapy (CBT) has been recognized as effective for individuals with AN; however, its limited accessibility due to a variety of factors presents a significant challenge (Duggan et al. 2025). In the United Kingdom, a mere 27.4% of women experiencing eating disorders received adequate treatment over their lifespan (Micali et al. 2017). In Japan, the estimated rate of CBT implementation was around 6.2% (Takahashi et al. 2018). The idea of delivering CBT self‐help programs implemented on websites via the internet (ICBT) continues to be developed with the aim of improving the availability of CBT. Therapists often utilize email, phone, or chat tools to support their patients after each session, and such interventions are referred to as guided ICBT (Andersson 2014). Guided ICBT for mental disorders has demonstrated promising results over the past two decades (Andersson et al. 2019).

The current evidence suggests that guided ICBT for AN, when delivered with therapist guidance, can effectively reduce eating disorder‐related behaviors (Hamatani et al. 2022) and help increase body mass index (BMI) in the short term (Fichter et al. 2012). However, the long‐term outcomes of guided ICBT for AN remain unclear. The study by Fichter et al. (2013) has been the sole research that has reported follow‐up data for guided ICBT, with findings limited to mid‐term outcomes at 9 months. Fichter et al. (2013) found that patients who completed nine sessions‐maintained BMI gains and showed favorable prognosis. However, the study focused on patients receiving specialized inpatient care for eating disorders in Germany; thus, the applicability of this evidence to outpatient settings and other cultural contexts may be limited.

To make an informed decision about adopting a new intervention technique, assessing both its therapeutic efficacy and its cost‐effectiveness is crucial. Guided ICBT is considered to be more cost‐effective than traditional face‐to‐face CBT (Hedman et al. 2012) because it requires limited therapist resources, with sessions often lasting less than 10 min per patient per week (Andersson 2009; Barak et al. 2009). However, there is no study that has investigated the cost‐effectiveness of guided ICBT for AN.

The objective of this study was to investigate the long‐term outcomes and cost‐effectiveness of adding guided ICBT to outpatient treatment for AN, drawing upon both our own data and publicly available data from Japan.

## 2 Methods

### 2.1 Study Design

This study involved a 3‐year follow‐up of women with AN who had participated in guided ICBT during a previous single‐arm trial (Hamatani et al. 2022). The current follow‐up study protocol was approved by the Institutional Review Boards of Chiba University (HJK0072‐24) and the University of Fukui (20240120). See the Supporting Information for the intervention.

### 2.2 Participants

The eligibility criteria in the previous trial were: (a) meeting the *Diagnostic and Statistical Manual of Mental Disorders*, Fifth Edition (DSM‐5) diagnostic criteria for AN (American Psychiatric Association 2013); (b) being aged 15–65 years; (c) receiving standard treatment; (d) having no plans to change medications or start new treatments during the study period; (e) having access to telecommunications equipment to use the ICBT program; (f) possessing the necessary information and communication technology skills; and (g) not having received CBT in the last 2 years (Hamatani et al. 2022).

### 2.3 Procedures

A total of twelve women with AN who had received and completed a guided ICBT intervention in a single‐arm trial conducted in Japan were invited to participate in this follow‐up study (Hamatani et al. 2022). Of these twelve participants, follow‐up data were obtained from eleven. To foster engagement, a handwritten note of appreciation was included with each mailed questionnaire. If no response was received, up to two postal reminders were sent, followed by an email reminder. Upon receiving the completed questionnaires, “thank you” emails were sent to the participants. Some participants included personal notes in the envelopes returned, such as “thank you” messages or updates on their current situation. All questionnaires were self‐administered, and the outcome measures are described in detail in the following sections. No additional study‐provided ICBT or other psychotherapy was delivered during the follow‐up period, while usual care continued. All data were collected by the first author (S.H.).

### 2.4 Outcome Measure

The global Eating Disorder Examination Questionnaire (EDE‐Q) score (Fairburn and Beglin 1994; Mitsui et al. 2017) and BMI were set as outcomes to evaluate the severity of AN. The EDE‐Q assesses attitudes and behaviors related to eating disorders experienced in the past 28 days. It consists of 22 items rated on a 7‐point Likert scale ranging from 0 (never/not at all) to 6 (daily/extremely), yielding four subscale scores: restraint, shape concern, weight concern, and eating concern. The global score represents the mean of the four subscale scores, with higher scores indicating greater eating disorder symptomatology; the scores on these subscales constituted the secondary outcomes (further details below). The reliability and validity of the Japanese version of the EDE‐Q have been confirmed (Mitsui et al. 2017). We defined clinical remission based on a previous study, using a cutoff score of 1.57 on the total EDE‐Q score; this cutoff value, supported by a sensitivity of 0.897 and a specificity of 0.901 for potential AN, must be the optimal threshold reported following receiver operating characteristic analysis of patients with AN and healthy controls (Meule et al. 2024). BMI is a measure used to estimate if an individual's weight is healthy relative to their height. BMI can be calculated by dividing their weight (in kilograms) by the square of their height (in meters). According to the BMI‐based severity specifiers for AN, ≤ 17 kg/m^2^ is mild, 16–16.99 kg/m^2^ is moderate, 15–15.99 kg/m^2^ is severe, and < 15 kg/m^2^ is extreme (American Psychiatric Association 2022). Additional secondary outcome measures included the Body Checking Questionnaire (BCQ) as a measure of body‐checking behaviors, the Body Shape Questionnaire (BSQ) as a measure of body shape concerns, the Patient Health Questionnaire‐9 (PHQ‐9) as a measure of depressive symptoms, and the Generalized Anxiety Disorder‐7 (GAD‐7) as a measure of anxiety symptoms, with details presented in the Supporting Information.

To evaluate the cost‐effectiveness of guided ICBT, we calculated quality‐adjusted life years (QALYs) using the EuroQol 5‐Dimension 5‐Level (EQ‐5D‐5L) questionnaire (Tsuchiya et al. 2002; van Hout et al. 2012; Ikeda et al. 2015). The EQ‐5D‐5L is a generic health‐related quality‐of‐life (QOL) measure that assesses five dimensions: mobility, self‐care, usual activities, pain/discomfort, and anxiety/depression. Each dimension is scored on a five‐level scale ranging from “no problems” to “extreme problems.” QALY scores range from 0.00 (dead) to 1.00 (perfect health). The EQ‐5D‐5L has demonstrated strong reliability and validity across diverse populations and clinical conditions, including mental and physical health disorders, in international studies (Herdman et al. 2011; Janssen et al. 2013; Buchholz et al. 2018).

### 2.5 Statistical Analysis

We conducted descriptive statistics on the epidemiological data and characteristics of the participants in this follow‐up investigation. A one‐way repeated‐measures analysis of variance (ANOVA) was conducted as the primary analysis. In addition, post hoc comparisons were performed to determine whether there was a significant decrease in the outcomes at each assessment point compared with the pre‐assessment point. These comparisons were conducted using Fisher's least significant difference (LSD) method, which does not include adjustment for multiple comparisons. No correction for multiple comparisons was applied owing to the exploratory nature of the study. All the analysis was conducted using SPSS version 29 (IBM Corp., Armonk, New York, United States), with a significance level set at *p* < 0.05.

The cost‐effectiveness analysis was performed to evaluate the provision of additional guided ICBT to outpatients with AN receiving standard care. We performed cost‐effectiveness analysis using an incremental cost‐effectiveness ratio (ICER), which has been widely used in health economic evaluations (Drummond et al. 2015). The ICER, defined as the incremental cost divided by the incremental effect, is a measure developed to assess the cost‐effectiveness of medical interventions. It is calculated using the following formula:

$$\mathrm{ICER}=\frac{\Delta \mathrm{Cost}}{\Delta \mathrm{Effect}}$$

Incremental costs were determined based on the total costs of guided ICBT. The guided ICBT clinician costs were estimated based on face‐to‐face CBT rates in Japan, as specific rates for guided ICBT clinicians are not currently defined. According to the Ministry of Health, Labour and Welfare (2020) in Japan, the fee for a CBT session exceeding 30 min ranges from JPY 3500 (for nurses) to JPY 4800 (for physicians). Given that our guided ICBT sessions lasted 15 min, consistent with previous research (Lenhard et al. 2017), we estimated guided ICBT clinician costs to be approximately half of those of CBT. Maintenance costs included a monthly platform fee of JPY 1200 and a monthly chat‐tool fee of JPY 6600, which were estimated based on service usage for 3 months. The per‐participant guided ICBT maintenance cost was calculated based on the number of participants. The platform utilized was Wix (Wix.com Ltd., Tel Aviv, Israel) and the chat tool was ShareMedical (ShareMedical Inc., Tokyo, Japan). The incremental effect was calculated as the difference between the area under the curve (AUC) of QALYs based on EQ‐5D‐5L QOL values at each assessment point and the AUC of QALYs assuming no change in QOL over 3 years (benchmark), in accordance with previous studies (Matsumoto et al. 2020, 2024).

## 3 Results

### 3.1 Participants’ Characteristics

**FIGURE 1:**
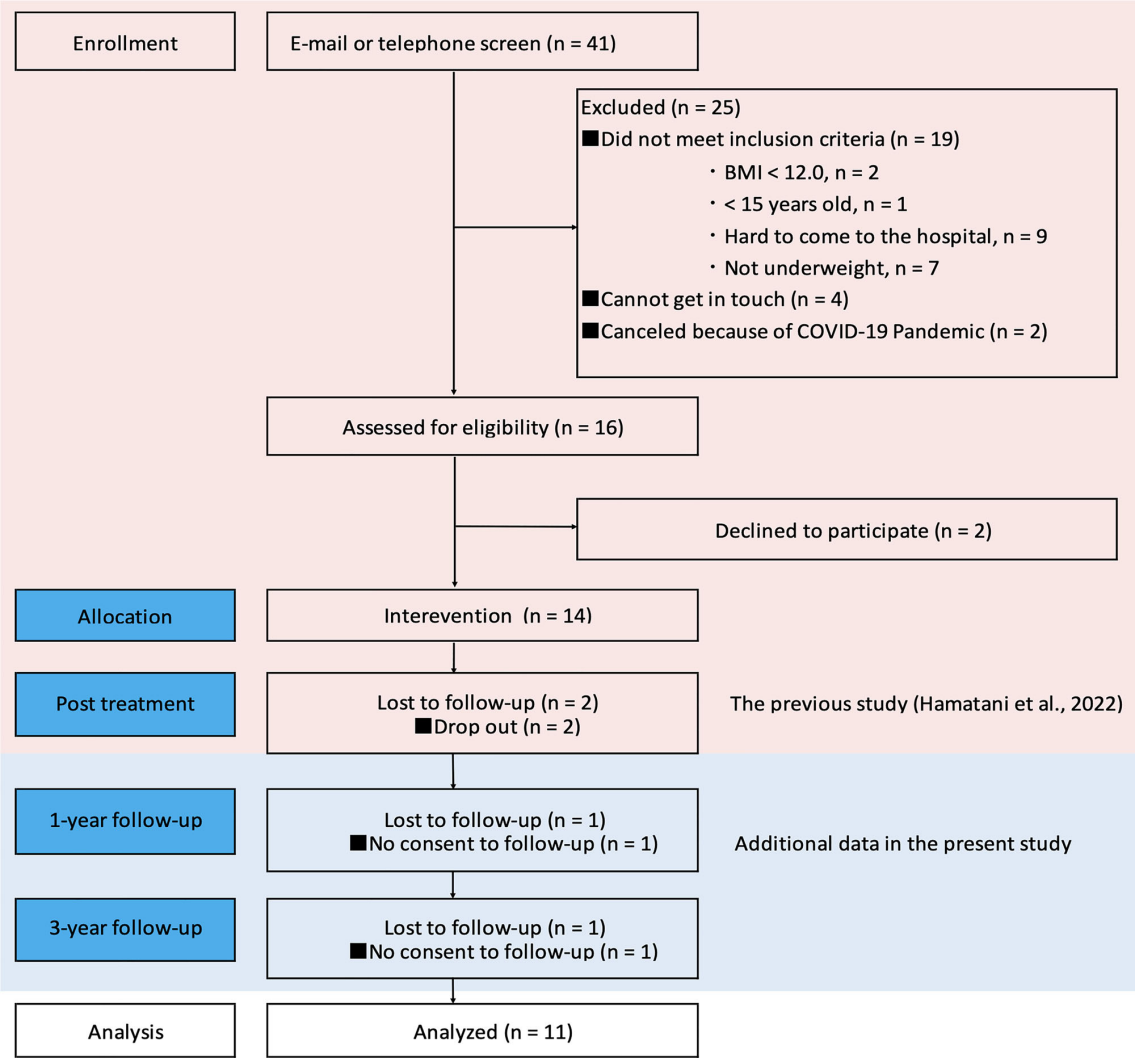
Participant flow in the 3‐year follow‐up study.

Figure 1 shows the current study flowchart. Eleven of them returned the self‐report questionnaires at both the 1‐ and 3‐year follow‐up assessments (response rate: 91.7%). The typical participants were in their late 20s and female, with an average of approximately 14 years of education, although some were still in high school at baseline. Demographic data of the participants are presented in Table 1. There were no reports of changes in outpatient treatment policies.

**TABLE 1:** Demographic data and clinical characteristics.

Demographics		
	Age in years, mean (SD)	27.8 (9.1)
	Years of education, mean (SD)	14.1 (3.1)
	Estimated IQ using the JART, mean (SD)	111.1 (7.8)
	AQ total score, mean (SD)	20.9 (8.8)
	ASRS‐total score, mean (SD)	2.4 (2.5)
Comorbidities, *n* (%)		
	Major depressive disorder, *n* (%)	3 (27.3)
	Generalized anxiety disorder, *n* (%)	2 (18.2)
AN subtype, *n* (%)		
	Binge eating/purging, *n* (%)	7 (63.6)
	Restricted, *n* (%)	4 (36.4)

Abbreviations: AN, anorexia nervosa; AQ, Autism‐Spectrum Quotient; ASRS: Adult ADHD Self‐Report Scale; JART, Japanese Adult Reading Test.

### 3.2 Long‐Term Outcomes

**FIGURE 2:**
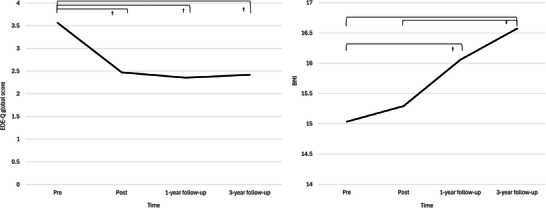
Trajectories of mean and 95% confidence intervals for EDE‐Q global score and body mass index.

Figure 2 illustrates the trends in mean of the EDE‐Q global score and BMI. Table 2 shows a significant decrease in the severity of AN. The EDE‐Q global score significantly decreased from pre‐intervention to all post‐intervention assessment points, with effect sizes ranging from Cohen's *d* = −0.70 to −0.65. BMI significantly increased from pre‐intervention to the 1‐year and 3‐year follow‐up points, and also significantly increased from immediately post‐intervention to the 3‐year follow‐up point (Figure 2). The remission rate was 36.4% (*n* = 4/11) immediately after treatment, 45.5% (*n* = 5/11) at 1‐year follow‐up, and 45.5% (*n* = 5/11) at 3‐year follow‐up.

**TABLE 2:** Mean (SD) and ANOVA results with effect size estimates.

Outcome	Assessment time point	Mean (SD)	*F* value	Mean difference from baseline (95% confidence interval)	*p* value	Cohen's *d*
EDE‐Q, global score			5.81	—	0.003	
	Baseline	3.56 (1.60)			—	—
	Post‐treatment	2.47 (1.53)		−1.09 (−1.95, −0.23)	0.018^a^	−0.70
	1‐year follow‐up	2.35 (1.76)		−1.21 (−2.08, −0.34)	0.011^a^	−0.72
	3‐year follow‐up	2.42 (1.90)		−1.14 (−2.16, −0.13)	0.031^a^	−0.65
Body mass index			5.45	—	0.014	
	Baseline	15.03 (1.39)	—	—	—	—
	Post‐treatment	15.29 (1.48)	—	0.259 (−0.12, 0.63)	0.154	0.18
	1‐year follow‐up	16.06 (1.68)	—	1.02 (0.12, 1.93)	0.030^a^	0.66
	3‐year follow‐up	16.57 (1.73)	—	1.54 (0.29, 2.79)	0.020^b^	0.98

*Note*: A negative Cohen's *d* value signifies a reduction in psychological symptoms, whereas a positive value indicates an increase in body mass index (BMI).

Abbreviation: EDE‐Q; Eating Disorder Examination Questionnaire.

^a^ *p* < 0.05 significant difference in pairwise comparisons with baseline score.

^b^ *p* < 0.05 significant difference in pairwise comparisons with both baseline and post‐treatment scores.

### 3.3 Cost‐Effectiveness of Guided ICBT

**FIGURE 3:**
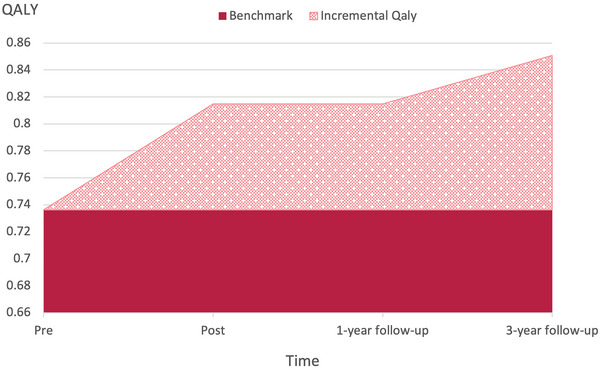
Incremental quality‐adjusted life years (QALYs) over the 3‐year follow‐up period.

Figure 3 displays QALYs over 3 years after guided ICBT. QALYs significantly increased from pre‐intervention to both post‐intervention (mean difference [MD] = 0.08, 95% confidence interval [CI]: 0.01–0.15, *p* = 0.39, Cohen's *d* = 0.67) and the 3‐year follow‐up (MD = 0.12, 95% CI: 0.01–0.22, *p* = 0.35, Cohen's *d* = 1.00), respectively. The incremental QALYs over the 3‐year period were 0.2714, and the incremental costs were 30,263 Japanese Yen. The ICER calculated using these estimates was JPY 111,507; this was calculated using the exchange rate of 154 JPY/USD as of December 17, 2024, for a total of USD 724.

## 4 Discussion

### 4.1 Principal Findings

This study investigated the long‐term outcomes—up to 3 years—of guided ICBT for women with AN in Japanese outpatient psychiatric settings. In the long‐term evaluation, AN severity decreased and BMI increased. The estimated ICER was JPY 111,507 (USD 724), which was below the Japanese willingness‐to‐pay threshold of JPY 5 million per 1.00 QALY (Fukuda and Shiroiwa 2019). These results provide preliminary support for the potential of guided ICBT as a practical and promising option for improving long‐term outcomes in patients with AN receiving outpatient care.

### 4.2 Comparing to Previous Work

The current study suggests that guided ICBT holds promise for the long‐term treatment of AN in outpatient. This is consistent with prior work in Germany that examined mid‐term outcomes (Fichter et al. 2013). The German study reported that at 9 months, mean BMI (SD) increased from 18.0 (1.3) to 19.5 (2.9) in participants who completed all 9 sessions, and from 17.8 (1.3) to 18.4 (2.6) in those who completed 8 or fewer sessions. These increases in BMI are broadly in line with the outcomes we observed, despite our study population having a lower baseline BMI (mean 15.0 [SD 1.4] to 16.6 [SD 1.7]), indicative of more severe AN. Taken together, this evidence suggests that guided ICBT may be effective in the long term. To our knowledge, this 3‐year follow‐up study is the longest to examine the long‐term effects of guided ICBT for AN, extending previous knowledge on guided ICBT for AN.

Despite the substantial increases in BMI, from a baseline mean (SD) of 15.03 (1.39) to 16.02 at 1 year and 16.54 at 3 years, the mean BMI remained below the normal range. The mean weight at baseline was 37.5 kg, increasing to approximately 39.7 kg and 41.1 kg at the 1‐year and 3‐year follow‐ups. Here, we highlight the need for cautious interpretation; given the chronic nature of the condition, a proportion of patients continue to meet the diagnostic criteria for AN. However, the significant BMI increase, with effect sizes ranging from moderate to large (Cohen's *d* = 0.66 to 0.98), and consistent improvements over time suggest that guided ICBT can facilitate sustained enhancements in patient outcomes—a trend not typically observed in short‐term interventions (Hamatani et al. 2022). Because ICBT programs are web‐based, users can review the treatment content even after the intervention period. The iterative nature of guided ICBT, a specialized form of CBT, may contribute to these sustained therapeutic effects (Matsumoto et al. 2024).

The threshold for the willingness‐to‐pay for 1.000 QALY in Japanese people has been JPY 5 million (Fukuda and Shiroiwa 2019). In our result for cost‐effectiveness, the willingness‐to‐pay for guided ICBT was JPY 111,507 (USD 724 at 154 JPY/USD), which is far below the threshold in Japan. This study is the first in the world to evaluate the cost‐effectiveness of guided ICBT. The results are consistent with those of a Dutch cohort study on face‐to‐face CBT (van den Berg et al. 2022), and those current evidence suggests that CBT may be cost‐effective for AN outpatients. Nonetheless, the evidence provided by the current research was estimated based on a limited sample size and the utilization of some publicly available data; therefore, a careful interpretation of the findings is warranted. To estimate a true value for the cost‐effectiveness of specific treatments for AN, a treatment database should be constructed for people with AN, and cost‐effectiveness analysis should be conducted using a model that utilizes real‐world data. The cost‐effectiveness of guided ICBT for AN cannot yet be generalized based on the results of this study; however, our findings constitute the only available evidence within the Japanese National Health System and could inform decision‐makers.

### 4.3 Limitations

This study has several limitations. These include the small sample size, changes in social and treatment circumstances during the follow‐up period, and the absence of a control group. The small sample size may threaten the generalizability of the current findings. It is unclear whether significant changes occurred in the participants’ social factors and treatment approaches. The lack of a control group also makes it difficult to infer the superiority of guided ICBT. On the other hand, it is ethically and humanely problematic to withhold a promising treatment from patients with a severe mental illness like AN over a long period. Therefore, future research should compare the outcomes of patients receiving guided ICBT with those receiving other treatments—such as supportive psychotherapy—through the accumulation of real‐world data (Wilson and Booth 2024).

## 5 Conclusion

We observed AN severity was reduced in outpatients with AN at time of 3 years after guided ICBT. Although interpretation of these results should be cautious due to limitations such as the small sample size, our analyses suggest guided ICBT may be promising in terms of long‐term efficacy and cost‐effectiveness.

## Author Contributions

**Sayo Hamatani**: conceptualization, investigation, funding acquisition, writing – original draft, methodology, validation, visualization, writing – review and editing, project administration, formal analysis, data curation. **Kazuki Matsumoto**: writing – review and editing, conceptualization, investigation, formal analysis, data curation, methodology, validation, writing – original draft. **Jumpei Takahashi**: conceptualization. **Eiji Shimizu**: conceptualization. **Yoshiyuki Hirano**: conceptualization, data curation, resources. **Yoshifumi Mizuno**: supervision, conceptualization, resources, writing – review and editing.

## Conflicts of Interest

The authors declare no conflicts of interest.

## Peer Review

The peer review history for this article is available at https://publons.com/publon/10.1002/brb3.70731.

## Supporting information




**Supporting Information**: brb370731‐sup‐0001‐SI.pdf

## Data Availability

The data that support the findings of this study are openly available in OSF at https://osf.io/37qnu/?view_only=24089217d2254e98b33a65686c6744ea.
