# Supplementary material for: Long‐Term Outcomes of Guided Internet‐Based Cognitive Behavioral Therapy for Anorexia Nervosa: 3‐Year Follow‐Up
Source: Brain Behav. 2025 Sep 8;15(9):e70731. doi: 10.1002/brb3.70731 (PMC12417561; doi:10.1002/brb3.70731)
Supplement: Supplementary file 1 — Supporting Information: brb370731‐sup‐0001‐SI.pdf [file BRB3-15-e70731-s001.pdf]

Supplementary Material

Original paper

Long-term outcomes of guided internet-based cognitive behavioral therapy for anorexia nervosa: 3-year follow-up

Sayo HAMATANI, Kazuki MATSUMOTO, Jumpei TAKAHASHI, Eiji SIMIZU, Yoshiyuki HIRANO, Yoshifumi MIZUNO

Index

The guided internet-based cognitive behavioral therapy in the previous trial *1*

Secondary outcomes *1*

The subscales of the Eating Disorder Examination Questionnaire (EDE-Q) 1

Body Checking Questionnaire (BCQ) 2

Body Shape Questionnaire (BSQ) 2

Patient Health Questionnaire-9 (PHQ-9) 3

Generalized Anxiety Disorder-7 (GAD-7) 3

Results of Secondary Outcomes Over the 3-Year Follow-Up *3*

S Table 1. Results for Secondary Outcomes in Analysis of Variance *5*

References *9*

The guided internet-based cognitive behavioral therapy in the previous trial

In the previous trial, participants received guided access to a 3-month ICBT program via a chat tool operated by a therapist. The program comprised 12 treatment modules, with the first half of the modules incorporating techniques focused on metacognition (for details, see Hamatani et al., 2022). The content covered psychoeducation, cognitive bias modification, body image and attention training, exposure and response prevention, and relapse prevention, and was delivered in both video and text formats. Participants completed one module per week at a convenient time and received individualized feedback from a CBT therapist (one of the researchers) via the chat tool after completing each module. The feedback primarily included encouragement, answers to questions, and advice related to future homework assignments. The ICBT program was available during the initial phase of the trial but was no longer accessible after the trial ended and was not available during the follow-up period. Participants were notified by email before their access to the program was terminated and were prompted to download the explanation sections and PDF worksheets for each the CBT technique.

Secondary outcomes

The subscales of the Eating Disorder Examination Questionnaire (EDE-Q)

The EDE-Q assesses attitudes and behaviors related to eating disorders experienced in the past

28 days. The EDE-Q consists of 22 items rated on a 7-point Likert scale ranging from 0 (never/not at all) to 6 (daily/extremely), yielding four subscale scores: restraint, shape concern, weight concern, and eating concern (Fairburn & Beglin, 1994; Mitsui & Komaki, 2017). These subscale scores are derived from the mean of the scores for the questions related to each theme.

Body Checking Questionnaire (BCQ)

The BCQ is a 23-item self-report questionnaire developed to assess body checking behaviors. It consists of three correlated subfactors: overall appearance checking, checking of specific body parts, and idiosyncratic checking rituals. The BCQ has demonstrated good test-retest reliability ($r = .94$), and strong concurrent and discriminant validity (Reas et al., 2002).

Body Shape Questionnaire (BSQ)

The BSQ is a 34-item self-report scale developed to evaluate concerns and dissatisfaction regarding body shape over the previous four weeks. Each item is rated on a 6-point scale (1 = never to 6 = always), and total scores reflect the degree of body shape concern, with higher scores indicating greater dissatisfaction. The original version has demonstrated high internal consistency and validity (Cooper et al., 1987), and the Japanese version has shown similarly good psychometric properties (Mera et al., 2011).

Patient Health Questionnaire-9 (PHQ-9)

The PHQ-9 is a self-report questionnaire designed to screen for and assess the severity of depressive symptoms. It comprises nine items that assess the frequency of depressive symptoms during the preceding two weeks. Total scores are used to determine the severity of depression (Spitzer et al., 1999; Muramatsu et al., 2018). The Japanese version of the PHQ-9 has demonstrated good internal consistency (Cronbach's $\alpha = .87$) and validity in both clinical and general populations (Muramatsu et al., 2018).

Generalized Anxiety Disorder-7 (GAD-7)

The GAD-7 is a self-report questionnaire used to screen for and assess the severity of anxiety symptoms (Spitzer et al., 2006; Muramatsu, 2014). It comprises seven items that assess the frequency of anxiety symptoms during the previous two weeks. Total scores are used to determine the severity of general anxiety symptoms. The Japanese version of the GAD-7 has shown high internal consistency (Cronbach's $\alpha = .92$) and good construct validity (Muramatsu, 2014).

Results of Secondary Outcomes Over the 3-Year Follow-Up

In addition to the improvements in BMI and the EDE-Q global score, several secondary outcomes showed favorable trends. Significant long-term improvements were observed for the EDE-Q subscales of restraint, shape concern, and weight concern, with moderate to large effect sizes (Cohen's $d = -.53$ to $-.84$). The BCQ scores also significantly decreased over time ($d = -.67$), suggesting a reduction in

body-checking behaviors. While the BSQ scores improved moderately, they did not reach statistical significance. Regarding comorbid symptoms, both the PHQ-9 and GAD-7 scores decreased immediately after treatment. However, at the 1-year and 3-year assessments, despite the effect sizes being moderate, no significant differences were observed ($d = -.51$ to $-.68$). Improvements in QOL were also observed, with EQ-5D-5L scores significantly increasing from baseline to 3-year follow-up ($d = 1.00$).

S Table 1. Results for Secondary Outcomes in Analysis of Variance

Outcome	Time point	Mean (standard deviation)	*F*-value	Mean difference (95% confidence interval)	*p*-value	Cohen's *d*
EDE-Q, restraint subscale			6.77	-	.001	
	Baseline	3.89 (2.01)	-	-	-	-
	Post-treatment	2.93 (2.00)	-	−.96 (−2.10, .17)	.087	−.48
	1-year follow-up	2.09 (2.26)	-	−1.80 (−2.82, −.78)	.003‡	−.84
	3-year follow-up	2.44 (2.14)	-	−1.46 (−2.52, −.39)	.012†	−.70
EDE-Q, eating concern subscale			2.38	-	.089	
	Baseline	3.05 (1.69)	-	-	-	
	Post-treatment	1.87 (1.49)	-	−1.18 (−2.08, −.29)	.015†	−0.74
	1-year follow-up	2.16 (1.87)	-	−.89 (−1.96, .18)	.094	−.50
	3-year follow-up	2.38 (1.92)	-	−.67 (−2.02, .67)	.291	−0.37
EDE-Q, shape concern subscale			4.94	-		

	Baseline	3.78 (1.50)	-	-	-	-
	Post-treatment	2.51 (1.52)	-	−1.28 (−2.19, −.36)	.011†	−.85
	1-year follow-up	2.64 (1.69)	-	−1.15 (−2.07, −.23)	.019†	−.72
	3-year follow-up	2.53 (2.17)	-	−1.25 (−2.28, −.22)	.022†	−.67
EDE-Q, weight concern subscale			5.42	-	.004	
	Baseline	3.53 (1.87)	-	-	-	
	Post-treatment	1.96 (1.55)	-	−1.56 (−2.56, −.56)	.006†	−.91
	1-year follow-up	2.53 (1.94)	-	−1.00 (−1.95, −.05)	.041†	−.53
	3-year follow-up	2.33 (2.16)		−1.20 (−2.15, −.25)	.018†	−.59
Body Checking Questionnaire			4.72	-	.008	
	Baseline	69.09 (31.03)	-	-	-	-
	Post-treatment	57.45 (26.98)	-	−11.64 (−24.32, 1.04)	.068	−.40
	1-year follow-up	55.73 (31.32)	-	−13.36 (−23.81, −2.92)	.017†	−.43
	3-year follow-up	49.82 (26.28)	-	−19.27 (−35.04, −3.51)	.021†	−.67

Body Shape Questionnaire			2.79	-	.058	
	Baseline	113.91 (41.84)	-	-	-	
	Post-treatment	91.00 (40.24)	-	−22.91 (−47.44, 1.62)	.064	−.56
	1-year follow-up	95.09 (40.88)	-	−18.82 (−42.19, 4.56)	.103	−.45
	3-year follow-up	91.64 (49.35)	-	−22.27 (−51.50, 6.96)	.120	−.49
Patient Health Questionnaire-9			2.34	-	.093	
	Baseline	13.27 (5.95))	-	-	-	-
	Post-treatment	9.73 (5.08)	-	−3.55 (−7.04, −.05)	.047†	−.64
	1-year follow-up	10.27 (5.52)	-	−3.00 (−8.14, 2.14)	.223	−.52
	3-year follow-up	9.00 (6.51)	-	−4.27 (−8.81, .27)	.062	−.68
Generalized Anxiety Disorder-7			2.12	-	.118	
	Baseline	10.27 (5.41)	-	-	-	-
	Post-treatment	6.00 (3.90)	-	−4.27 (−7.17, −1.37)	.008†	−.91
	1-year follow-up	7.82 (4.19)	-	−2.46 (−7.66, 2.75)	.318	−.51
	3-year follow-up	7.18 (6.01)	-	−3.09 (−7.72, 1.54)	.168	−.54

EuroQol 5-Dimension 5-Level questionnaire			2.46	-	.082	
	Baseline	0.74 (0.12)	-	-	-	-
	Post-treatment	0.81 (0.11)	-	.08 (.01, .15)	.039†	.67
	1-year follow-up	0.81 (0.12)	-	.08 (−.05, .20)	.188	.65
	3-year follow-up	0.85 (0.11)	-	.12 (.01, .22)	.035†	1.00

Note. † $p < .05$ significant difference in pairwise comparisons with baseline score. ‡ $p < .05$ significant difference in pairwise comparisons with both baseline and post-treatment scores. Abbreviations: EDE-Q; Eating Disorder Examination Questionnaire. A negative Cohen's d value signifies a reduction in psychological symptoms, whereas a positive value indicates an increase in quality of life (QOL).

References

- Cooper PJ, Taylor MJ, Cooper Z, Fairburn CG. The development and validation of the body shape questionnaire. *Int J Eat Disord*. 1987;6(4):485-494. doi:10.1002/1098-108X(198707)6:4<485::AID-EAT2260060405>3.0.CO;2-O
- Hamatani S, Matsumoto K, Takahashi J, et al. Feasibility of guided internet-based cognitive behavioral therapy for patients with anorexia nervosa. Internet Interv. 2022;27:100504. doi:10.1016/j.invent.2022.100504
- Fairburn CG, Beglin SJ. Assessment of eating disorders: interview or self-report questionnaire? Int J Eat Disord. 1994;16(4):363-370. doi:10.1002/1098-108X(199412)16:4<363::AID-EAT2260160405>3.0.CO;2-%23
- Meule A, Hilbert A, de Zwaan M, Brähler E, Koch S, Voderholzer U. Cutoff scores of the Eating Disorder Examination-Questionnaire for the German population. Int J Eat Disord. 2024 Mar;57(3):602-610. doi: 10.1002/eat.24133. Epub 2024 Jan 22. PMID: 38258314.
- Mitsui T, Yoshida T, Komaki G. Psychometric properties of the eating disorder examination-questionnaire in Japanese adolescents. *Biopsychosoc Med.* 2017;11:9. doi:10.1186/s13030-017-0094-8Muramatsu et al., 2018
- Muramatsu K, Miyaoka H, Kamijima K, Muramatsu Y, Tanaka Y, Hosaka M, Miwa Y, Fuse K, Yoshimine F, Mashima I, Shimizu N, Ito H, Shimizu E. Performance of the

Japanese version of the Patient Health Questionnaire-9 (J-PHQ-9) for depression in primary care. Gen Hosp Psychiatry. 2018 May-Jun;52:64-69. doi: 10.1016/j.genhosppsych.2018.03.007.

- Muramatsu K. Patient Health Questionnaire (PHQ-9, PHQ-15) Japanese version and Generalized Anxiety Disorder-7 Japanese version up to date. Stud Clin Psychol. 2014;7:35-39.
- Spitzer RL, Kroenke K, Williams JB. Validation and utility of a self-report version of PRIME-MD: the PHQ primary care study. *JAMA*. 1999;282(18):1737-1744. doi:10.1001/jama.282.18.1737
- Spitzer RL, Kroenke K, Williams JB, Löwe B. A brief measure for assessing generalized anxiety disorder: the GAD-7. *Arch Intern Med.* 2006;166(10):1092-1097. doi:10.1001/archinte.166.10.1092.
- Reas DL, Whisenhunt BL, Netemeyer R, Williamson DA. Development of the body checking questionnaire: a self-report measure of body checking behaviors. *Int J Eat Disord*. 2002;31(3):324-333. doi:10.1002/eat.10012
